# “Grabbing” autonomy when the learning environment doesn’t support it: an evidence-based guide for medical learners

**DOI:** 10.12688/mep.19182.1

**Published:** 2022-06-17

**Authors:** Adam Neufeld

**Affiliations:** 1 Academic Family Medicine, University of Calgary, Calgary, Alberta, T3E 5S4, Canada

**Keywords:** autonomy, medical learner, basic psychological needs, self-determination, well-being

## Abstract

According to self-determination theory (SDT), environments which assist the basic psychological needs for autonomy, competence, and relatedness will facilitate autonomous motivation, learning, and wellness. On the other hand, environments which introduce external controls and power dynamics into the equation will do the opposite. Educational studies support these principles, yet most have focused on learners’ autonomy satisfaction as a passive process (e.g., via support or hindrance by educators), rather than the agentic pursuit that SDT emphasizes. In this commentary, I draw on my experience as a senior resident and SDT researcher and focus on how medical learners can “grab” more autonomy when the learning environment does not support it. I present a hypothetical case of a preceptor whose teaching style is controlling and unfortunately well-known to medical learners. I then unpack the case and outline different strategies that medical learners can use to navigate this type of interpersonal conflict.

## Introduction

### The controlling preceptor: a hypothetical case

Please imagine that you are a senior medical learner about to start your Adult Emergency Medicine rotation. Today’s shift is from 16:00-23:00 with a preceptor, M.L., who you have not worked with before. Nervous but excited, you make your way to the Emergency Department (ED), where this preceptor greets you. They briefly ask which year you are in, and if there is anything you want to focus on, which leads you to believe they are interested in teaching and getting to know you. Unfortunately, the next 7 hours does not unfold this way…

The preceptor is nice to others, but with you, they are standoffish, impatient, and intimidating. Instead of encouraging you and providing structured guidance, independence, and choices, they scrutinize your work, limit you to seeing only new patients, and assign them to you without discussion: no re-assessments, entering of orders, following of investigations, calling consultants, or handling of discharges. Whenever you see a patient together, they also repeat your history and physical exam and exclude you from the conversation. They often pressure you with rapid-fire questions, use a harsh tone of voice, and dismiss your ideas and opinions. Your initial motivation is quickly replaced by anxiety about assessment, and it undermines your performance. In the end, your preceptor gives you a negative evaluation in public, and provides no affirmation for what you did well. You finish the shift feeling frustrated and stressed about the formal evaluation to come.

### Unpacking the case from a self-determination theory point of view

This is a classic example of a preceptor whose style was controlling, counterproductive, and psychologically harmful ^1, 2^. They were demanding and disrespectful, and instead of adopting an unconditional positive regard, which still permits them to challenge and provide corrective feedback to learners ^3, 4^, they used a harsh tone of voice and punished the learner for making mistakes. They also provided no encouragement for what they did well. Instead of feeling excited about their learning, the preceptor made the learner feel pressured and incapable of being their true self. This led to feelings of alienation and anxiety, and the learner was primed to fear mistakes and to not let their preceptor down ^1, 5^. Having to submit a mandatory evaluation to this preceptor, combined with being micromanaged and distrusted, ultimately undermined the learner’s autonomy and performance ^1, 5^. In the end, the preceptor’s feedback was destructive, and the learner ended their shift feeling annoyed, deflated, and worried about the future.

Does this scenario seem familiar? If so, you are not alone. This kind of preceptor behaviour is unfortunately still alive and well in medicine, and unintentional as it may be, it can be seriously harmful to medical learners. I, myself, have encountered this situation many times throughout my medical training, and through trials and tribulations, have learned a thing or two about how to deal with it. Part of this discovery was learning about self-determination theory (SDT) and how its universal needs framework could help me maintain my own motivation and wellness, regardless of what was happening around (or to) me. The following section focuses on some of these aspects and how we can all adapt, as learners, to make the most of these negative situations. Note: this paper is geared towards medical learners; however, I also invite medical educators to consider this article, as they play a critical role here, and they too are lifelong learners who share the same human needs.

### Practical tips for dealing with controlling learning climates

Now some preceptors are just demanding and rigid in their ways, so trying to quickly adapt and incorporate their feedback should first be attempted, provided it does not compromise one’s values and integrity. To negotiate a more motivationally supportive environment for oneself, suggesting a change in the flow of instruction from the preceptor is also an option. Reeve and Tseng ^6^ refer to this as “agentic engagement” and explain how working intentionally with the teacher like this can “pull” more autonomy support out of them. If tensions are felt to be too great, however – between what is best for one’s learning and wellness versus what is best for achieving a desirable evaluation – know that this is true-to-life and there are other steps we can take to satisfy our need for autonomy. SDT’s view is that “asserting” autonomy (fighting against the obstacles that prevent us from expressing our opinions, interests, and desires) is a natural response to need-thwarting environments ^7, 8^. Thus, one may already be doing aspects of this in one’s journey towards becoming a physician. The aim here is to make the implicit explicit, so as to facilitate that process.

***Recognize the situation.*** First, knowing when the learning climate is “controlling” and what that concept means is helpful because naming the problem allows us to process it better ^9^. Part of this is recognizing when and how the environment is hindering our sense of autonomy, competence, and relatedness. Research in SDT shows that authority figures tend to be controlling for two main reasons – either because of external pressures they are facing, which frustrate their own basic psychological needs, and/or because they believe that controlling others with incentives and pressures (i.e., out of “mustivation”) is a better, easier way to motivate them than trying to inspire them and support their autonomy (i.e., out of “wantivation”) ^10^. Ironically, being autonomy-supportive is a skill that anyone can learn ^11^, it has many reciprocal benefits for the teacher ^12^, and it promotes better learning, performance, and wellness for medical learners, as well as quality of patient care ^1, 2, 13^.

***Lean into negative emotions.*** Second, understanding this preceptor’s frustrating behaviour and how it would undermine anyone’s performance, recognising it is psychologically harmful and counterproductive, and that it is generally not the learner’s fault, are also key realizations. According to SDT, facing and trying to process these thoughts and feelings, rather than suppressing them or allowing them to overwhelm us, is a more autonomous and healthy approach to coping ^14^. This is especially true when our emotions are challenging or painful because it allows for an inward reflection that fosters self-awareness and self-acceptance ^15, 16^. It can also help to quieten our ego and promote self-compassion when our performance falls short of expectations ^17^. Medical programs often teach socioemotional matters like mindfulness and resilience ^18, 19^. However, from my experience, they seldomly discuss or apply them in the context of controlling learning climates and frustrations around basic psychological needs, which are both strong predictors of motivation, learning, and well-being outcomes in medicine ^20^.

***Self-reflect and put things into perspective.*** Third, it is useful to remind oneself that this is a single shift, with a single preceptor, out of many hundreds or more in one’s medical training. In other words, consider the subjectivity of evaluations, and the inherent personality factors that play into them. Here, it is also helpful to compare this preceptor and their assessment(s) to others one has worked with. To ask oneself if things are representative, off base, or if we one is simply having a bad day, which happens to everyone. It is also valuable to consider how certain preceptor styles will set us up to succeed or fail before we even begin. For example, the above preceptor limited the learner to only seeing new patients, whose issues were undifferentiated and arguably the hardest part of working in the ED. If the same preceptor had let the learner take part in the ongoing care of their patients and encouraged them to take more responsibility for them, they would have better supported the learner’s autonomy, learning, and well-being ^1, 21^. Either way, studies in SDT show that mindfulness and autonomy-supportive self-talk can help us to deal with this kind of situation ^22, 23^. Negative life experiences are also great opportunities for learning. Our integration versus defensiveness of them simply depends on whether we can meet our basic psychological needs or not ^7, 24– 26^.

***Connect with patients and peers.*** Fourth, cultivate relationships with patients. They are presenting because they are unwell and the care and support that we can provide them is often rewarding and psychologically fulfilling ^27^. Take the time that is afforded to get to know them. Collaborate with the nursing staff and others. When a preceptor is controlling, distant, and/or unsupportive, this is a sure way to “grab” one’s need for relatedness, which supports our autonomy ^28^. Patients, allied health staff, and other medical colleagues also tend to take notice of these efforts, and it can come back around to positively shape a preceptor’s impressions and behaviours. While I do not recommend disrespecting your staff or venting to patients, taking the time to sit with and comfort them, and to express one’s thoughts and feelings, can go a long way – both for them and for us, as medical learners ^7, 27^. Of course, if there are other medical students or residents nearby who one can confide in and talk to, take that opportunity for social and emotional support as well. Provided that exchanges are socially appropriate and constructive, this too can help us claim a little more need satisfaction and confidence when we need it ^29, 30^.

***Stay curious and remember the big picture.*** Fifth, there is nothing preventing us from staying up to date on what is occurring with our patients, and from watching out for certain investigations (e.g., labs or imaging) to come back, based on our initial assessments. This can help to close the loop and promote our learning, grit, and self-determination ^7^. Depending on what was agreed upon at the beginning of one’s shift (e.g., “everything goes for ordering tests except d-dimers, CT, or MRI”), one may also consider ordering new investigations if they are felt to be relevant. This shows a priority of patient care that can help to positively reframe the situation ^31^, and to (re)shift one’s perceived locus of causality (i.e., felt autonomy for behaviour) from external back to internal ^23^. A limitation here is that some preceptors may perceive this kind of behaviour as overly assertive, so being cautious and using one’s better judgment is recommended in this case.

***Find closure through honest and constructive feedback.*** Finally, when one’s formal evaluation gets completed, there will typically be the chance to provide some feedback in return. Tempting as it may be to denigrate the preceptor, use this opportunity to diplomatically share one’s negative experience with the medical program instead, including who the preceptor was and how their style of supervision undermined one’s learning, performance, and wellness. Consider qualifying the feedback with specific examples, along with ways that the preceptor could improve, to help facilitate better collaboration, engagement, and learning outcomes in the future. Ultimately, the preceptor is doing what they think is best (even if it is inefficient and psychologically harmful) and taking the time to recognize this, and that they are also learners, shows maturity, empathy, and integrity. According to SDT, altruistic behaviour like this can also help us to gain some closure and satisfy our basic psychological needs ^32^.

## Conclusions

As a medical learner, one will inevitably find oneself in controlling learning environments that can lead to feelings of being pressured, powerless, and frustrated. One may not be able to change the situation or the preceptor, but there are cognitive, emotional, and behavioural strategies that can be used to adapt and maintain one’s motivation and wellness. Guided by SDT, the aim of this commentary was to discuss some of these strategies, and how to use them, when the learning climate is not supportive of autonomy. Identifying the controlling environment, proactively engaging with one’s preceptor, using mindfulness and self-talk, connecting with patients and peers, taking personal responsibility for one’s learning, and giving constructive feedback to the program. These actions can facilitate integration of the negative experience, support one’s clinical learning and development, and help one to meet their basic psychological needs.

## Data availability

No data are associated with this article.
