# Supplementary material for: “Grabbing” Autonomy When the Learning Environment Doesn’t Support it: An Evidence-based Guide for Medical Learners
Source: MedEdPublish (2016). 2023 Jul 10;12:43. Originally published 2022 Jun 17. [Version 2] doi: 10.12688/mep.19182.2 (PMC10450255; doi:10.12688/mep.19182.2)
Supplement: Peer review history

# Peer review: Reviewer response for version 2

**DOI:** 10.21956/mep.21118.r34060

**Published:** 2023-08-24

**Authors:** Lucas M. Jeno

**Affiliations:** 1 University of Bergen, Bergen, Norway

I approve the revisions made to this manuscript.

If evidence from practice is presented, are all the underlying source data available to ensure full reproducibility?

Not applicable

Is the topic of the practical tips discussed accurately in the context of the current literature

Partly

Are all factual statements correct and adequately supported by citations?

Yes

Are the conclusions drawn balanced and justified on the basis of the presented arguments?

Partly

Are arguments sufficiently supported by evidence from the published literature and/or the authors’ practice?

Yes

Reviewer Expertise:

Self-Determination Theory, higher education, learning, achievement, autonomy-support

I confirm that I have read this submission and believe that I have an appropriate level of expertise to confirm that it is of an acceptable scientific standard.

# Peer review: Reviewer response for version 1

**DOI:** 10.21956/mep.20549.r33373

**Published:** 2023-06-09

**Authors:** Lucas M. Jeno

**Affiliations:** 1 University of Bergen, Bergen, Norway

Thank you for the opportunity to review this commentary entitled ““Grabbing” autonomy when the learning environment doesn’t support it: an evidence-based guide for medical learners“. This is an interesting manuscript. However, before being suitable for indexing, the manuscript need some expansions and revisions.

 I appreciate the brevity of the manuscript, although the paper is on how to motivate one self, the manuscript focuses a lot on the context. The focus of the manuscript is on controlling (need-thwarting) instructors. The focus could have easily been on a student with low motivation for completing a task, not mentioning the context. The reason why I highlight this is because the context exemplified here is very need-thwarting I would say. If actually this is the case that the average student would thrive independent of such a controlling context over time, more citations on similar studies are needed, or a more thorough description. I would recommend re-focusing the story here because although autonomy orientations (personality) predict wellness and thriving, this effect is usually always mediated by need-satisfaction (usually provided by the context).

 Having said this, there are some additional “tips” that are missing from this manuscript. For one, I think the concept of need-crafting deserves to be mentioned because this is basically what the manuscript is about. Some recent papers by Laporte et al are recommended. There are also some papers by Koestner, and Cheon on setting self-set intrinsic goals, which also I encourage to consider in these tips ^1^^,^^2^^,^^3^^,^^4^^,^^5^. Sheldon have papers on reflecting on ones relative autonomous motivation (organismic valuing process), which is think is also very interesting in terms of practical suggestions. I real strength in this paper is the focus on mindfullness for the integrative process. I guess emotional regulation could be mentioned more explicitly, especially if the author continues with a focus on the context? Another strength is the broad review of important literature in such a short manuscript. While I'm not familiar with the tone of this journal and the level of formality, my comments should obviously be evaluated in light of this, but I do believe the manuscript would be strengthened if a less context-situation is provided in the intro, and more additional SDT tips are provided.

If evidence from practice is presented, are all the underlying source data available to ensure full reproducibility?

Not applicable

Is the topic of the practical tips discussed accurately in the context of the current literature

Partly

Are all factual statements correct and adequately supported by citations?

Yes

Are the conclusions drawn balanced and justified on the basis of the presented arguments?

Partly

Are arguments sufficiently supported by evidence from the published literature and/or the authors’ practice?

Yes

Reviewer Expertise:

Self-Determination Theory, higher education, learning, achievement, autonomy-support

I confirm that I have read this submission and believe that I have an appropriate level of expertise to confirm that it is of an acceptable scientific standard, however I have significant reservations, as outlined above.

## References

[1] : Adolescents as active managers of their own psychological needs: The role of psychological need crafting in adolescents' mental health. *J Adolesc* .2021;88: 10.1016/j.adolescence.2021.02.004 67-83 10.1016/j.adolescence.2021.02.004 33662706

[2] : The role of daily need crafting in daily fluctuations in adolescents’ need-based and affective experiences. *Motivation and Emotion* .2022;46(2) : 10.1007/s11031-021-09921-2 137-149 10.1007/s11031-021-09921-2

[3] : In search of the organismic valuing process: the human tendency to move towards beneficial goal choices. *J Pers* .2003;71(5) : 10.1111/1467-6494.7105006 835-69 10.1111/1467-6494.7105006 12932212

[4] : Autonomous motivation, controlled motivation, and goal progress. *J Pers* .2008;76(5) : 10.1111/j.1467-6494.2008.00519.x 1201-30 10.1111/j.1467-6494.2008.00519.x 18705645

[5] : A Self-Determination Theory Perspective on How to Choose, Use, and Lose Personal Goals.2018; 10.1093/oxfordhb/9780199841332.013.39 10.1093/oxfordhb/9780199841332.013.39

# Peer review: Reviewer response for version 1

**DOI:** 10.21956/mep.20549.r33168

**Published:** 2023-05-30

**Authors:** Kenneth Masters

**Affiliations:** 1 Medical Education and Informatics Department, College of Medicine and Health Sciences, Sultan Qaboos University, Muscat, Oman

This is an interesting article offering guidance for medical learners in an adversarial clinical setting.

 The tone of the article is a little more colloquial than one would normally find in an academic paper, but, given that it is aimed directly at the learner, that is probably appropriate.

 I have only one concern, and that is a small issue around the language used in the opening part of the paper:

- While I appreciate the need for non-specific pronouns, when both parties in a conversation use the same pronoun, the author needs to be extremely careful.  Given that this is a hypothetical case, the author has greater freedom than if a real case were being used. Please consider either using different pronouns or repeat the nouns, otherwise, a sentence like this requires several readings, and the reader becomes so bogged down in trying to understand who is who, that the message is lost: “They also provided no encouragement for what they did well.” 
- In fact, in a later sentence, the grammatical structure of the sentence implies that the “their” in the opening subordinate clause refers to “the preceptor” in the main clause, making the sentence barely intelligible “Instead of feeling excited about their learning, the preceptor made the learner feel pressured and incapable of being their true self.”  At this stage, I think several readers would be tempted to simply give up.

So, a good and useful read, but a bit of clarity required in the opening scenario.

If evidence from practice is presented, are all the underlying source data available to ensure full reproducibility?

Not applicable

Is the topic of the practical tips discussed accurately in the context of the current literature

Yes

Are all factual statements correct and adequately supported by citations?

Yes

Are the conclusions drawn balanced and justified on the basis of the presented arguments?

Yes

Are arguments sufficiently supported by evidence from the published literature and/or the authors’ practice?

Yes

Reviewer Expertise:

Medical Informatics

I confirm that I have read this submission and believe that I have an appropriate level of expertise to confirm that it is of an acceptable scientific standard.
